# An interpretation of intraosseous perfusion physiology and the effect of steroids

**DOI:** 10.1186/s40634-020-00251-9

**Published:** 2020-05-16

**Authors:** Michael Beverly, David Murray

**Affiliations:** Nuffield Department of Orthopaedics, Rheumatology & Musculoskeletal Sciences, University of Oxford, Botnar Research Centre, Nuffield Orthopaedic Centre, Headington, Oxford, OX3 7LD UK

## Background

This study was undertaken to explore the physiology of intraosseous perfusion and steroids. Intraosseous pressure (IOP) has been studied for at least 70 years but is poorly understood and has proved to be of limited clinical value [1, 2]. Intraosseous pressure has generally been assumed to be due to a venous back pressure or an intrinsic tissue turgor or pressure [3]. Several authors have reported difficulty in establishing a normal value for IOP [4], but generally IOP has been found to be raised in bone pain and osteonecrosis [5]. Corticosteroids are recognised to be associated with the development of osteonecrosis, and are thought to act by causing intraosseous fat cell swelling [6, 7].

Experimental models for osteonecrosis have included a variety of different animals and birds [8, 9]. The rabbit has frequently been used as a steroid treated model for osteonecrosis [10].

Our previous work showed that IOP may have underlying pulse and respiratory waves and a pulse pressure or difference between diastolic and systolic pressure, (PP) which is proportional to the IOP [11]. Here our primary aim was to use IOP to explore the physiology of bone perfusion, with the hypothesis that changes in proximal vascular occlusion would be of interest. We then used the same method but in a steroid treated group.

## Method

IOP was measured in the subchondral cancellous bone of the upper tibia of 21 (7 male, 14 female) New Zealand White rabbits (with ethical approval by the Royal Postgraduate Medical School under Home Office licence ELA 24/4994, Wellcome Trust Grant 12,425/1.5/SC). Methyl prednisolone 80 mg (Sigma Aldrich, Dorset, UK) was administered intravenously to six animals (1 male, 5 female) 3 weeks before the experiment. This gave a large dose of widely dispersed long acting corticosteroid.

The rabbits were anaesthetized through an ear vein with intravenous Sublimaze (fentanyl, Janssen-Cilag Pty Ltd., NSW 2113, Australia), 2 ml of 0.05 mg/ml solution depending on the size of the animal. Anaesthesia was maintained with Valium (diazepam), (Genentech Inc., San Francisco, CA 94080), 0.5 ml of 5 mg/ml solution alternating with Sublimaze (fentanyl) 0.5–1.0 ml given slowly on an approximately ½ hourly basis. A saline filled 23G venesection needle was pushed into the upper tibial bone percutaneously by rocking the needle through a 5 to 10-degree arc along the line of the bevel of the needle to penetrate the cortex. The needles were placed in the approximate tibial centre within a few millimeters of the subchondral surface. The needles were connected by saline filled lines to pressure transducers (Druck PDCR75, Druck Ltd., Leicester, UK) and to a four-channel chart recorder (Lectromed MX4P-31, Lectromed Ltd., Jersey, Channel Islands). The transducers were calibrated on a 0–100 mmHg scale and zeroed before each run. Vascular clips were placed on the exposed proximal femoral artery and vein alternately. The subsequent change in IOP and PP was recorded after 30 s.

The aorta was opened immediately postmortem and 200 ml diluted 50% barium sulphate suspension in water (Microcat 50 mg/ml, Guerbet Laboratories Ltd., Solihull, UK) was infused by hand. The femora were dissected out, fixed in 10% formalin and decalcified over a period of 72 h in 10% nitric acid. The tibiae were not used as they might have had damage or leakage artefacts from the IOP needles. Colour photographs were made of the femora. Angiograms were carried out using fine grain film at 40 kV, 100 mA, 100 cm tube-target distance with a 20 s exposure.

A series of experiments were undertaken to investigate:

1. Variations in basal IOP (IOPb) and the associated basal PP (PPb) at rest in all 21 subjects.
2. The effect of proximal arterial (IOPa) and proximal venous (IOPv) occlusion and the delta or subtraction difference (IOPv-IOPa) in all subjects. We assumed that the difference was a measure of perfusion at the needle tip.
3. The effect of steroids on these parameters in the six steroid treated subjects.
4. Decalcification, photography and micro angiography of the femora.

### Statistical method

Because anaesthetic and experimental duration varied from 30 min to 2 h, an average of the early, middle and late values was used for each needle site basal IOPb. At the same times the associated pulse pressure (PP) or the difference between the top and bottom of the pulse trace was recorded and averaged. After each vascular occlusion, one reading of IOP and PP at 30 s was made.

Results were expressed as means, standard deviations and ranges. Normality was not formally tested but we considered that Student’s t-test was sufficiently robust to determine if there were significant differences. When each subject was used as its own control, paired t-tests were used. Otherwise unpaired t-tests were used. The Pearson test was used to determine correlations with *p* < 0.05 considered to be statistically relevant.

## Results

### 1 Variation in IOP at rest

There were 7 males and 14 females. Their weights were similar, male average = 4305 g (SD 81 g, range 3760–4680 g), female average = 4491 g (SD 144 g, range 2640–5560 g), t-test *p* = 0.4.

**Fig. 1:**
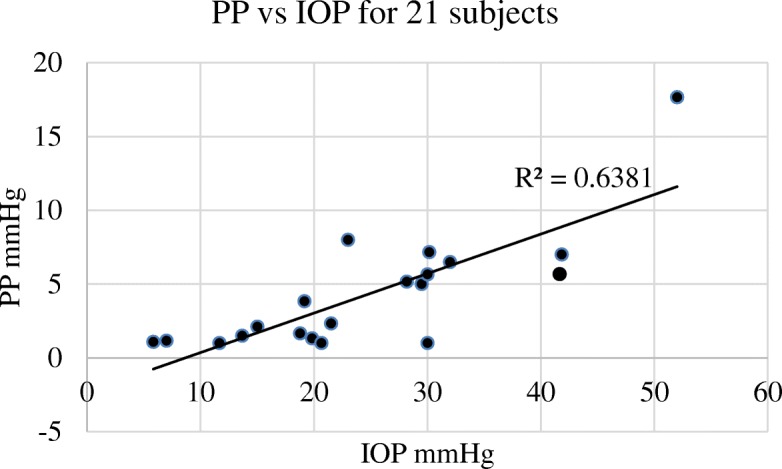
Showing the relationship between basal IOP and basal PP. IOP is proportional to the PP, Pearson correlation *R*^*2*^ = 0.6381

The basal IOPb varied considerably between the 41 sites tested among 21 subjects with a mean of 24.8 mmHg (SD 11.6 mmHg) and an associated basal PP (PPb) of 4.3 mmHg (SD 3.9 mmHg). PP was correlated with IOP (*R*^*2*^ = 0.64) as in Fig. 1.

### 2 Proximal vascular occlusion

**Fig. 2:**
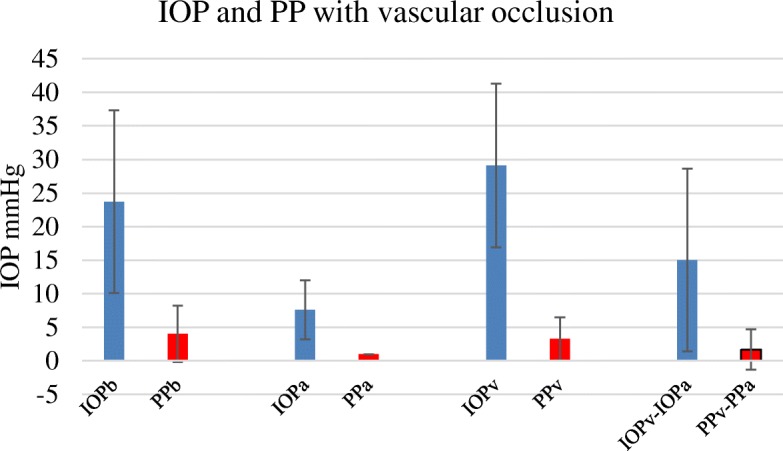
For 21 subjects (41 sites) Fig. 2 shows the initial or basal pressure, IOPb and PPb, the arterial clamp pressure, IOPa and PPa, the venous clamp pressure, IOPv and PPv and the perfusion difference, (IOPv-IOPa) and PPv-PPa. IOPb to IOPa and IOPb to IOPv were significantly different *p* < 0.0001. Error bars bars SD. Blue – IOP, Red – PP

In 21 subjects with 41 records proximal arterial occlusion IOPa caused the pressure to fall from a basal IOPb of 24.8 mmHg (SD 11.6 mmHg) to IOPa of 7.7 mmHg (SD 4.6 mmHg, *p* < 0.0001) and PPb fell from 4.3 mmHg (SD 4.6 mmHg) to PPa of 1 mmHg (SD 0 mmHg, *p* < 0.0001). With proximal venous occlusion IOPv the pressure rose from 24.8 mmHg to an IOPv of 29.1 mmHg (SD12.2 mmHg), *p* < 0.0001 while PPv did not change significantly (3.4 mmHg, SD 3.3 mmHg, *p* = 0.41). The difference between the venous occlusion IOPv and arterial occlusion IOPa (IOPv-IOPa) was 15.1 mmHg, SD 10.1 mmHg (*p* < 0.0001) as in Fig. 2.

### 3 Steroid treatment

Five females and one male received steroids. After 3 weeks steroid treated subjects were visibly thinner and weighed less than the controls (3963 g vs 4611, t-test *p* < 0.003). Corticosteroids in this model at this dose appeared to cause cachexia with marked loss of muscle, body fat and weight.

**Fig. 3:**
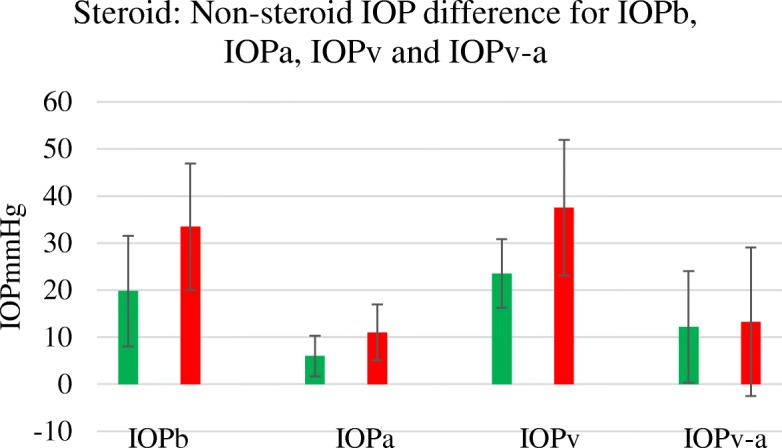
Steroid treated subjects had higher IOPb (*p* < 0.002), IOPa (*p* < 0.03) and IOPv (*p* < 0.05) pressure than the non-steroid treated controls. *N* = 6 steroid treated, 15 controls. The subtraction or perfusion values (IOPv-IOPa) remained similar (*p* = 0.6). Error bars SD

The six subjects treated with steroids had a raised IOPb averaging 33.5 mmHg (SD 13.5 mmHg), compared with the 15 controls with an IOPb of 19.6 mmHg (SD 2.9mHg), *p* < 0.002. The PPb in the steroid group was 6.9 mmHg (SD 6.1 mmHg) and in the controls was 2.9 mmHg (SD 2.6 mmHg), *p* < 0.004. The steroid treated venous occlusion value IOPv was 37.5 mmHg (SD14.4 mmHg) with a control venous occlusion IOPv value of 23.5mHg (SD 7.3 mmHg), *p* < 0.05). The steroid treated arterial occlusion value IOPa was 11 mmHg (SD 5.9 mmHg) with a control IOPa value of 6 mmHg (SD 4.3 mmHg), *p* < 0.03) as in Fig. 3. The difference in perfusion range at the needle tip determined by subtraction IOPv-IOPa remained relatively stable between the non-steroid treated group 12.2 mmHg (SD 11.9mHg) and the steroid treated group 13.3 mmHg (SD 15.8 mmHg), *p* = 0.6 as in Fig. 3.

### 4 Angiography

**Fig. 4:**
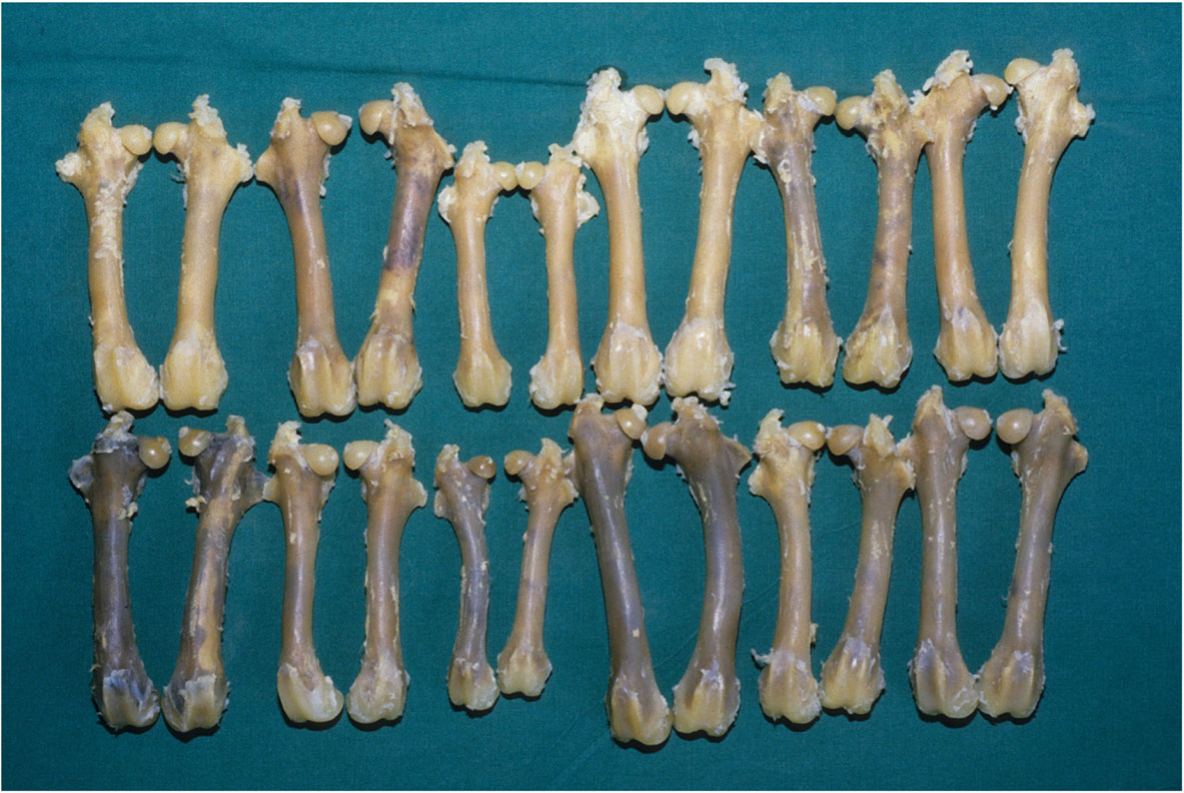
Top row steroid treated bones are whiter due to more barium retention. Bottom row controls are darker and contain less barium than the steroid treated femora

Diluted barium was injected down the aorta. The femora were removed, decalcified and photographed. The steroid treated femora were noticeably whiter than the controls as in Fig. 4. This appeared to be because they are better filled with barium as in the photographs.

**Fig. 5:**
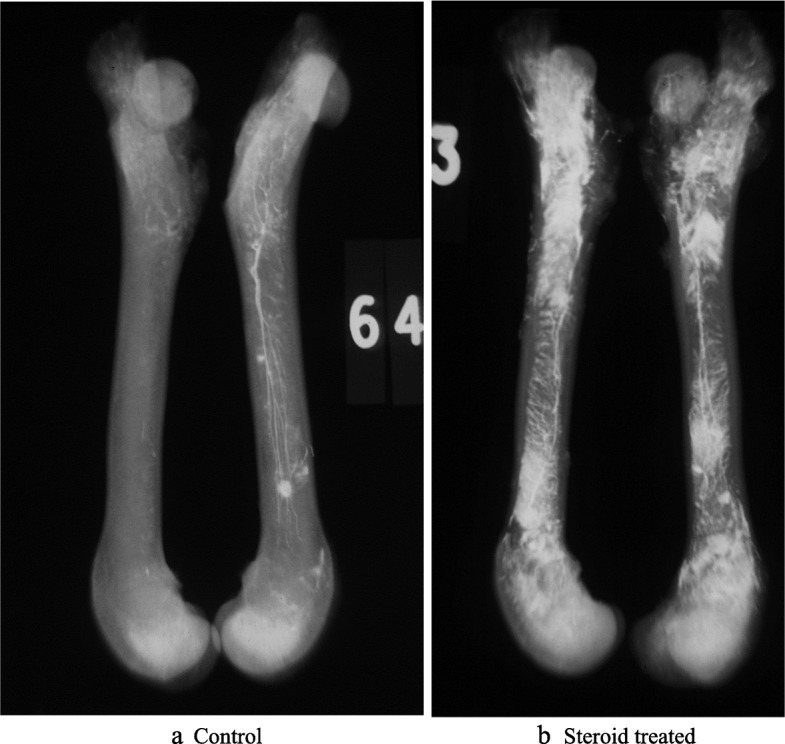
**a** and **b** showing examples of femoral angiogram microradiographs. The left radiograph 5a shows a pair of femora from a control barium angiogram. The right femoral pair 5b were from a steroid treated subject. After steroid treatment there was better barium filling

Microradiographs were carried out after decalcification. They appeared to show that the whiter steroid treated bones contained more barium than the controls as in Fig. 5.

## Discussion

We propose that IOP varies because of the variability in capillaries contacted by the needle tip. Our work demonstrates that, even with a repeatable technique, a wide range of IOP values is obtained by putting a needle into bone [12]. The spread of those values and their correlation with the associated PP supports the concept that the IOP is a reflection of local perfusion conditions in a small blood pool at the needle tip. We suggest that a higher IOP and PP are present where there is contact with an artery and a lower IOP if only fat and small blood vessels or veins are contacted. The significant fall in IOP with arterial occlusion (IOPb – IOPa 24.8 mmHg to 7.7 mmHg *p* < 0.0001) and the lesser rise in IOPb to IOPv (24.8 mmHg to 29.1 mmHg, *p* < 0.0001) with venous occlusion indicates that the majority of the recorded IOPb is due to the arterial supply side rather than being a venous back pressure.

For decades IOP has been measured in order to understand osteonecrosis and other diseases [13, 14]. Yet no other solid organ has had pressure measured in this way. Previous authors have usually considered IOP to have a static or constant value which was said to increase in osteonecrosis, with steroids and with bone pain. Many investigators have found IOP to be variable, so making measurement of IOP difficult to interpret [4].

The loss in both IOP and the pulse pressure wave with proximal arterial occlusion also demonstrates that most of the IOP and all the pulse pressure wave is due to arterial side supply pressure. Any residual pressure after clamping the proximal artery represents the true venous back pressure at the needle tip. Similarly, the increased IOP and preservation of the PP with proximal venous occlusion probably represents the best possible supply pressure obtainable at capillary level at that needle tip.

Any single IOP measurement is therefore of limited value whereas by subtracting IOPv – IOPa it is possible to obtain, for the first time, a useful idea of the perfusion pressure range obtainable at a needle tip deep in cancellous bone. Although subtraction has been used with imaging such as in digital angiography [15], we can find no previous description of this concept in perfusion studies.

Pulse pressure was taken as the difference between the top and the bottom of the IOP trace and was proportional to the absolute IOP as in Fig. 1. The subtraction difference between venous occlusion (IOPv) and arterial occlusion (IOPa) or IOPv-IOPa indicates the range of pressure obtainable at that individual needle tip. PP is therefore a variable which is usually dependent on the basal or initial IOPb. IOPv-IOPa is more an indication of the perfusion range obtainable at that needle tip. We would expect that in ischaemic bone, irrespective of the basal IOPb, the IOPv-IOPa difference, which is a measure of perfusion would be less than in healthy bone.

We were interested in the effect of steroids on IOP in a model which had previously been developed to explore perfusion physiology and the effects of loading on IOP [16]. The present study confirmed that steroid treatment raised IOP, as most previous authors have found [17]. The corticosteroid dose used in our model was one which caused cachexia and weight loss much like that seen in man with heavy corticosteroid use causing a type of diabetic keto-acidosis. The photographs and angiograms showed an increase in bone vascularity. In this model a large dose of steroid appeared to cause the intraosseous space to shrink, rather than the fat cells swelling, as described in other models. Here the bone appears ‘emptier’ after steroid treatment and this allowed better filling of the microvascular tree and therefore a better supply pressure or IOPb at the needle tip. There was an associated significant increase in the steroid treated pulse pressure. Importantly, the perfusion or IOPv-IOPa in the treated subjects did not reduce, as might be expected in ischaemic bone, but remained similar to that in healthy bone. In our model the already satisfactory perfusion in normal control bone did not improve further by shrinking the intraosseous volume. We remain unable to offer an explanation as to how corticosteroids cause osteonecrosis in other models or in man.

If, irrespective of the initial IOPb, there is little difference in IOPv-IOPa, it would be reasonable to suppose that the perfusion achievable at the needle tip is poor. Conversely and irrespective of the initial IOP, if there is a large ‘subtraction’ value it would be reasonable to suppose that at the needle tip good perfusion is attainable. We suggest that this new physiological subtraction approach could also be used clinically in other situations, for example, in compartment syndromes. If a catheter or needle in the affected calf muscle shows little change in pressure with proximal thigh tourniquet applications at venous or arterial occlusion pressure, then there is a poor perfusion at the catheter tip. A wider perfusion pressure range would indicate that there is better perfusion at the needle tip and that surgical intervention is not urgently required.

There are several possible limitations in this work. The subjects were of different gender, weights and ages. The control and steroid treated groups were of different sizes. IOP was measured in the proximal tibia for ease of access while the femora were used for the micro angiograms as the upper tibial IOP needles might have damaged the proximal tibia and distorted the angiograms. Needle placement was by hand to within a few millimeters’ accuracy only. Anaesthesia in these subjects was brittle and experimental duration varied. We did not obtain histology. We were unable to record blood pressure. Corticosteroids are known to raise systemic blood pressure and that may have caused a rise in IOP. Had that been the only cause, we would not have expected the visible barium and micro angiogram changes. Other possibilities exist, for example, that the pressure changes reflect flow through porous lacunocanalicular channels. We suggest that the observed speed of pressure change and visible pulsatility on traces excludes the possibility of pressure changes being the result of flow in micro channels. The sensitivity of the recording system may affect the pulse volume to a degree. The fluid or saline column within the needle from the blood pool up to the pressure transducer inevitably had a certain viscosity or inertia which might reduce the range seen in pulse pressure records.

## Conclusions

In a well-controlled experimental situation, we have identified substantial variability in IOP measurement. We suggest that this is because the needle records pressure only from the largest arteriole encountered in the blood pool at the tip. We have identified a new, complementary and perhaps more useful method of assessing bone perfusion by a subtraction technique with alternating proximal venous and arterial occlusion. This is demonstrated in our steroid model in which, although IOP rose, there was no significant change in perfusion. Our model differed from others with osteonecrosis as there appeared to be a reduced rather than increased intraosseous fat cell volume. Our study provides new insights into bone perfusion physiology. The subtraction technique may have useful clinical applications in bone and other areas.

## Data Availability

All data will be made available on request.
